# Sexual practices among unmarried adolescents in Tanzania

**DOI:** 10.1186/1471-2458-9-373

**Published:** 2009-10-06

**Authors:** Method R Kazaura, Melkiory C Masatu

**Affiliations:** 1 Epidemiology and Biostatistics, Muhimbili University of Health and Allied Sciences, Dar es Salaam, Tanzania; 2 Centre for Educational Development in Health Arusha, Arusha, Tanzania Department of Public Health, Tanzania

## Abstract

**Background:**

Sexual activities are increasingly changing from the cultural point of view what they used to be. Knowledge of these practices among adolescents may be a basis to create awareness among adolescents on practices that involve risks. This study aims to assess sexual practices among unmarried adolescents in Tanzania.

**Methods:**

A cross-sectional survey was conducted among in-school and out-of-school but unmarried adolescents aged 10 to 19 in five locations in Tanzania. A questionnaire was used to collect information and to characterize sexual practices among these adolescents.

**Results:**

About 32% of adolescents reported being sexually active; a higher proportion being males than females. The only inquired and reported sexual practices include vaginal sex, masturbation, oral and anal sex. About 15% of sexually active adolescents reported having multiple sexual partners. Significantly more males reported having multiple partners than females. Nearly 42% of sexually active adolescents reported having used a condom during most recent sexual act. Females reported older partners at first sexual act.

**Conclusion:**

Adolescents experience several sexual practices that include penetrative and non-penetrative. More males reported being sexually active than females. Despite adolescents reporting having multiple sexual partners, reported condom use during the most recent sexual act was low. We advocate for a more enhanced approach of reproductive health education that includes safer sex to adolescents without forgetting those in-schools.

## Background

Sexuality and sexual practices differ in context because of cultural and social environmental differences that exist in the society [1]. Although there is a great variability in sexual practices, particularly in sub-Saharan countries, such practices were always considered to go hand in hand with in taboos and cultures [2-4].

A tendency of increasing proportion of sexually active adolescents in Tanzania has been reported earlier [5,6]. Among the public health concerns are some of the reported types of sexual practices in the region that increase the risk for adverse health outcomes. A study conducted in Zimbabwe indicated that the most common type of sexual practice is penetrative vaginal sex and other forms were reported to be rare [7]. Although homosexual as well as heterosexual anal intercourse may have been practiced earlier in the continent, they are more being disclosed in recent times in different parts of Africa [8]. A recent study done in Kenya found that, apart from penile-vaginal intercourse, more than 40% of female sexual workers in Meru reported ever practising anal sex and 36.1% had experience of dry sex [9]. Available literature on sexuality and reproductive behaviour among adolescents in the region show that there have been minimal changes in risky sexual behaviours and their undesirable outcomes such as unwanted pregnancies, STIs and HIV [10-12].

In Tanzania, an increasing from about 20% to 50% in condom use during the past decade has been documented [5,13]. Nevertheless, risky sexual practices, for example, reported number of sexual partners in some parts of Tanzania has never levelled over time [14].

Other reported adolescents' sexual practices include heterosexual, sexual coercion and age differences between sexual partners [15-20]. Therefore, studies on sexuality and sexual practices among adolescents in sub-Saharan countries maybe one of the important stages to understand and fight against the outcomes risky sexual behaviours that may lead to unwanted pregnancies and sexually transmitted infections including HIV [21,22].

In this paper we use data generated from a survey on 'assessing risk sexual behaviour among adolescents' that was conducted in areas served by the Evangelical Lutheran Church of Tanzania. For the purpose of this paper, only data on sexuality and sexual practices have been used. Knowledge on types of sexual practices among adolescents may be useful in the design and implementation of appropriate measures towards improving adolescents' reproductive and sexual health.

## Methods

This cross sectional survey was part of baseline study for the planned Local Community Competence Building and HIV/AIDS Programme, to be based in five Dioceses of the Evangelical Lutheran Church in Tanzania (ELCT). These are Konde Diocese, South-Western, South Central, Iringa Diocese and Diocese in Mara Region. The selected Dioceses are characterized by high HIV prevalence ranging between 5.7 to 13.3% [23]. This study was conducted in the three villages in each Diocese. The target population for the study was adolescents (10 to 19 years) who were attending primary and secondary schools as well as those who were out-of-school. The estimated sample sizes for each study site were 400 primary schools, 100 secondary schools, and 200 out-of-school adolescents. In total we targeted about 2,500 in-school and 1,000 out-of-school adolescents.

For each Diocese, first, a random sample of about 133 primary schools was selected. Targets in each of the selected primary schools were students in class 5, 6 and 7. Since a class in a primary school consisted of several 'streams', these streams formed clusters. Therefore a cluster was selected at random in each class making three clusters per selected primary school. In total, 1567 students in primary schools were interviewed.

Second, another simple random sample consisted of 100 secondary schools in all five Dioceses. The target for secondary schools was students in forms 3 and 4. Like in primary schools, clusters consisted of 'streams' in each of form 3 and form 4. Then, a cluster was selected at random from which all students were interviewed. In total, there were 454 students from secondary schools. Classes 5 to 7 and forms 3 and 4 were believed to maximally capture the desired age of 10 to 19 years of students in schools.

Third, selection of out-of-school adolescents was done using cluster sampling technique. From each of the three simple randomly selected villages, three sub-villages were selected using simple random sampling. From each sub-village three "ten-cells" were also selected using simple random sampling. Ten-cells are the lowest administrative units in the Tanzania government structure. All households in the selected ten-cells were included in the sample and adolescents within these households were asked to participate in the study.

Out-of-school adolescents were interviewed at their household. Informed consent and permission to conduct interviews among dependant adolescents were sought from parents/guardians. While a self-administered questionnaire was used for in-school adolescents and filled in during school time, the out-of-school adolescents were interviewed at their household. This instrument has been used previously and its validity and reliability have been reported earlier [24]. The instrument was slightly modified to fit the settings, developed in English and then translated into Kiswahili, a language familiar to most Tanzanians.

Using the same data set collected for baseline information mentioned above, similar work has been published on risk sexual behaviour and associated factors [5]. For the purpose of this paper that aims at examining sexual practices, we used same data set but limiting ourselves to all in-school and only unmarried out-of-school adolescents (In Tanzania, primary and secondary schools students are not allowed to marry). Comparisons between categorical variables were performed using the Pearson's χ^2 ^test and one-way-ANOVA when comparing quantitative variables between groups.

Ethical clearance for the study was obtained from the National Institute for Medical Research. Permission to conduct the study was requested from the District Executive Directors and District Education Officers in each of the study sites as well as from local school and village authorities. Informed verbal consent to participate in the study was sought from respondents and assurance of confidentiality of the gathered information was given. Confidentiality was adhered to both among in and out-of-school adolescents when conducting interviews. None of the school teachers or parents/guardians was permitted to join the premises or to interfere with interviews.

## Results

A total of 2928 adolescents responded to the questionnaire. Of these, 2021 (69.0%) were in-school and the remaining 907 (31%) were out-of-school. Of the 907 out-of-school adolescents 747 (82.4%) who were unmarried are included in the analyses. Furthermore, we excluded 19 respondents because of missing key variables, thus remaining with 2749 respondents. Table 1 shows background information of the study sample.

**Table 1:** Background information of study sample (n = 2749)

**Characteristics**	**Number (%)**
Sample origin	
In-school	2021 (73.5)
Out-of-school	728 (26.5)
Sex	
Male	1419 (51.6)
Female	1330 (48.4)
Religion	
Christian	2527 (91.9)
Muslim	58 (2.1)
Other or no religion	164 (6.0)
Education*	
No formal	110 (4.0)
Some primary	2142 (83.6)
Above primary	310 (12.1)

* The total number in this category does not add up to 2749 because of non-responses

The mean age for all study participants was 15.3 (SD = 2.2) years. Males were significantly older than females; mean age for boys 15.6 (SD = 2.2) years and for females 15.0 (SD = 2.1); (F = 36.2, p < 0.01).

Table 2 shows types of sexual practices among study participants by gender. Although among all adolescents analyzed, 885 (32.2%) reported to be sexually active, adolescents were reporting more than one sexual practices (vaginal, anal, oral and masturbation) such that 125 (1109/885) sexual practices per 100 sexually active adolescents were reported. All (885) sexually active adolescents reported vaginal sex. Of all sexually active adolescents, 260 (29.4%) reported masturbation, 72 (8.1%) reported oral sex and 66 (7.5%) anal sex. There were significant differences of proportions between males and females reporting having vaginal intercourse and masturbation.

**Table 2:** Number (%) of sexually active adolescents reporting different sexual practices by sex (n = 885)

		**Sex**		
			
**Type of sexual practice**	**Total (%)**	**Male**	**Female**	**p-value**
Vaginal sex	885 (100)	605 (42.6)	280 (21.1)	< 0.01
Anal*	66 (7.5)	51 (8.5)	15 (5.4)	0.1
Masturbation	260 (29.4)	213 (35.6)	47 (17.0)	< 0.01
Oral	72 (8.1)	56 (9.4)	16 (5.8)	0.07

* All adolescents reporting anal sex also reported vaginal sex

Out of 885 sexually active adolescents, 141 (15.9%) reported to have had sex unwillingly. Reported reasons for sex against will were rape, 108 (12.2%), deception by the partner, 32 (3.6%) and one adolescent had sex because of custom pressures. More females, 76 (27.1%), reported to have been raped as compared to 32 (5.3%) males of all 280 and 605 sexually active female and male adolescents respectively.

The number of current sexual partners ranged from none to seven. Of 785 (100 sexually active adolescents did not respond to this question) reporting number of current sexual partners, 116 (14.8%) reported having multiple sexual partners. Significantly more males, 100 (18.8%), than females, 16 (6.3%), reported having multiple sexual partners (p < 0.01). Likewise, significantly more of the sexually active in-school adolescents, 82 (20.4%), reported having multiple sexual partners than their counterparts out-of-school adolescents, 34 (8.9%) (p < 0.01). The number of current sexual partners by sex and school status of respondents is indicated in table 3.

**Table 3:** Reported number of current sexual partners by sex and school status of sexually active adolescents (n = 785*).

**School status**	**Number of sexual partners**	**Females (%)**	**Males (%)**
In-school	None	20 (18.3)	42 (14.4)
	One	77 (70.6)	180 (61.6)
	Multiple	12 (11.0)	70 (24.0)
	Total	109 (100.0)	292 (100.0)
			
Out-of-school	None	47 (32.4)	57 (23.8)
	One	94 (64.8)	152 (63.6)
	Multiple	4 (2.8)	30 (12.6)
	Total	145 (100.0)	239 (100.0)

* 100 of the 885 sexually active adolescents did not respond to this question

**Figure 1:**
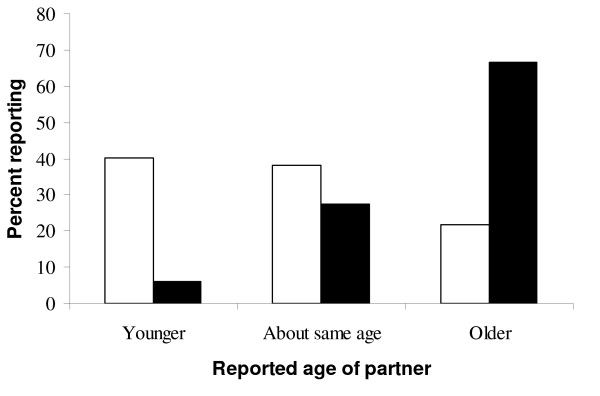
**Proportion of adolescents (by sex) reporting age of partner at first sexual intercourse (males = 575, females = 264)**. Black; Female. White; Male.

Females reported significantly older age of 14.5 (SD = 3.3) years at first sex than males, 13.0 (SD = 3.3) years (p < 0.01). Figure 1 compares age at first sex of respondent and reported age of the partner. One hundred and seventy six (66.7%) of girls reported sexual initiation with an older partner as compared to 124 (21.6%) boys among 264 and 575 sexually active girls and boys respectively who responded to this question.

Less than half, 42.0%, (371/885) of the sexually active adolescents reported use of a condom during the most recent sexual activity. Furthermore, of the 402 out-of-school sexually active adolescents, 190 (47.3%), reported using a condom during the most recent sexual act as compared to 181 (37.5%) of the 483 adolescents in-school (p < 0.01).

## Discussion

In Tanzania, like many other sub-Saharan African countries, the incidence of HIV among youths aged 15 - 24 years has been alarming. For example in 2005, HIV prevalence among this age group in South Africa accounted for 34% of all new infections [5]. The greatest known risky sexual practices associated with increased HIV incidence include unprotected penetrative sex, multiple sexual partners and young age at first sex [25].

In this study, more than 40% of adolescents reported to be sexually active. This proportion does not differ with the 2004 Tanzania estimate of premarital sex in the same age group [26]. But it is lower than the reported figures from Kisumu (Kenya) [27], and Rakai (Uganda) about 15 years ago [28].

Although very little is known about anal sex among adolescents and youths in sub-Saharan Africa and its contribution to HIV, self-reported anal sex in this study was about 8%. Nevertheless, since unprotected anal sex may be more risky for HIV transmission than unprotected vaginal sex (9), reproductive health education among adolescents should strongly stress on elevated risk for HIV and other viral infections among those having unprotected sex and especially unprotected anal intercourse.

Several studies have indicated that the risk of STIs including HIV increases with multiple sexual partners [29,30]. In this study, the proportion of adolescents reporting current multiple sexual partners is slightly higher than those reported from Zambia but within the limits of the national estimates [11,26].

Sexual coercion and rape have been previously reported in sub-Saharan African communities [15-18]. The magnitudes of reported sexual abuse in this study which range between 4 and 12 percent may have been under-reported since abuse is considered shameful hence remain secretive [31].

In our study, male reported younger partner and female reported older partner at first sexual debut. Despite this phenomena sounding common, to the ability of our knowledge, authors could not get any literature from Africa substantiating the idea. Additionally, in this study condom use among adolescents was less than 50%. Similar findings of low condom use have been previously reported in sub-Saharan African countries [32].

Previously, authors utilized this data set to explore predictors of risky sexual behaviours [5]. While the previous article dealt with a sample of in- and out-of-school adolescents, the present article excluded married out-of-school adolescents. On one hand, this may partly explain differences between the two articles with respect to number of sexual partners with respect to sample origin and on the other hand, some differences may be due to improved data cleaning.

The main limitation of this study is the accuracy of reported sexual practices among study participants. This could have happened in either by over- or under-reporting especially among in-school adolescents as suggested elsewhere [24]. In this study, we could not quantify and validate the magnitude of over- or under-reporting of experiences in sexual abuse. However, in a review involving several African countries, it was suggested that young women tend to under-report they ever had sex and males tend to report the opposite [33]. Furthermore, although in our study design we excluded lower (forms 1 and 2) secondary schools students among the selected strata, authors do not feel that such exclusion leads to potential bias. Yet, a complete simple random selection that includes all pupils in schools as a sampling frame may be tested to validate these findings.

## Conclusion

Unmarried adolescents in Tanzania experience several pre-marital sexual practices that include penetrative and non-penetrative. Female adolescents reported more of these sexual activities than males. Although 15% of sexually active adolescents reported having multiple sexual partners, yet reported current use of condom among these adolescents was low. Limited use of condom was more reported among in-school as compared to out-of-school adolescents. These findings suggest for a more rigor approach of reproductive health education and especially of safer sex to adolescents without forgetting those in-schools.

## Competing interests

The authors declare that they have no competing interests.

## Authors' contributions

MK conceptualized the problem. MM developed the protocol. MK and MM analyzed the data, drafted the paper and both authors contributed to the final manuscript.

## Pre-publication history

The pre-publication history for this paper can be accessed here:


